# Bilobed Gallbladder: A Rare Anatomical Variation Discovered During Laparoscopic Cholecystectomy

**DOI:** 10.7759/cureus.72300

**Published:** 2024-10-24

**Authors:** Alexis Arza, Erin Stitzlein, Jeremy Jen, Jin Y Park, Prashanth Ramachandra

**Affiliations:** 1 Department of Surgery, Drexel University College of Medicine, Philadelphia, USA; 2 Department of General Surgery, Mercy Fitzgerald Hospital, Darby, USA

**Keywords:** bilobed gallbladder, cholecystectomy, congenital, gallbladder, imaging, intraoperative

## Abstract

A bilobed gallbladder is a rare congenital anomaly with two lobes sharing a single cystic duct, typically diagnosed preoperatively and rarely identified intraoperatively. Only a small number of cases have been documented in medical literature with limited information on associated conditions.

A 22-year-old male patient, with a past medical history of cholelithiasis and no prior surgical history, presented with acute right upper quadrant pain and was diagnosed with acute cholecystitis. Initial ultrasound and magnetic resonance cholangiopancreatography imaging showed a distended gallbladder with multiple stones, but the bilobed gallbladder was only discovered during laparoscopic cholecystectomy, with an intraoperative retrospective imaging review confirming the diagnosis.

This anatomical variation can complicate surgery, particularly in achieving the critical view of safety. In this case, a dome-down approach was used to complete laparoscopic cholecystectomy without intraoperative or postoperative issues. Early and accurate diagnosis is challenging but crucial for successful management. In this report, we present our surgical approach to managing this patient. This report aims to contribute to the limited literature on bilobed gallbladders.

## Introduction

A bilobed gallbladder, a rare congenital anomaly, is defined as two gallbladder lobes with a single cystic duct [1-7]. This uncommon anatomical variation has fewer than 30 documented cases in existing literature [1,6], limiting the ability to determine its association with clinically significant risk factors such as cholecystitis, cholelithiasis, or choledocholithiasis. Additionally, there have been only four other cases that underwent laparoscopic cholecystectomy, and only a single case identified intraoperatively [6], with limited discussion within the literature regarding intraoperative diagnosis, imaging, and management. This case report aims to add to existing literature and describe surgical management of a bilobed gallbladder discovered intraoperatively.

## Case presentation

A 22-year-old male patient, with a past medical history of cholelithiasis and no prior surgical history, presented to the emergency department (ED) complaining of worsening pain in the right upper quadrant (RUQ) that began three days ago. He reports experiencing nausea and vomiting during meals, accompanied by fluctuating achy pain throughout the day. Upon arrival to the ED, the patient's vital signs were significant for elevated blood pressure at 150s over 90s, otherwise afebrile with normal vitals. Physical examination revealed RUQ tenderness with a positive Murphy’s sign. The abdomen was soft and non-distended, with no rebound tenderness or guarding. Initial laboratory results showed transaminitis, with aspartate transaminase at 188 U/L (reference range: 10-40 U/L), alanine transaminase at 565 U/L (reference range: 7-56 U/L), alkaline phosphatase at 134 U/L (reference range: 44-147 U/L), and total bilirubin at 5.7 mg/dL (reference range: 0.1-1.2 mg/dL).

A RUQ ultrasound displayed nonmobile gallstones that were impacted in a dilated cystic duct, along with gallbladder wall thickening and trace pericholecystic fluid. The common bile duct (CBD) was measured at 15mm. Due to elevated bilirubin and enlarged CBD, a magnetic resonance cholangiopancreatography (MRCP) was obtained, revealing a distended gallbladder containing multiple stones and an 8mm CBD with no choledocholithiasis. Upon diagnosis of acute calculus cholecystitis without choledocholithiasis, the patient was admitted and underwent laparoscopic cholecystectomy.

**Figure 1:**
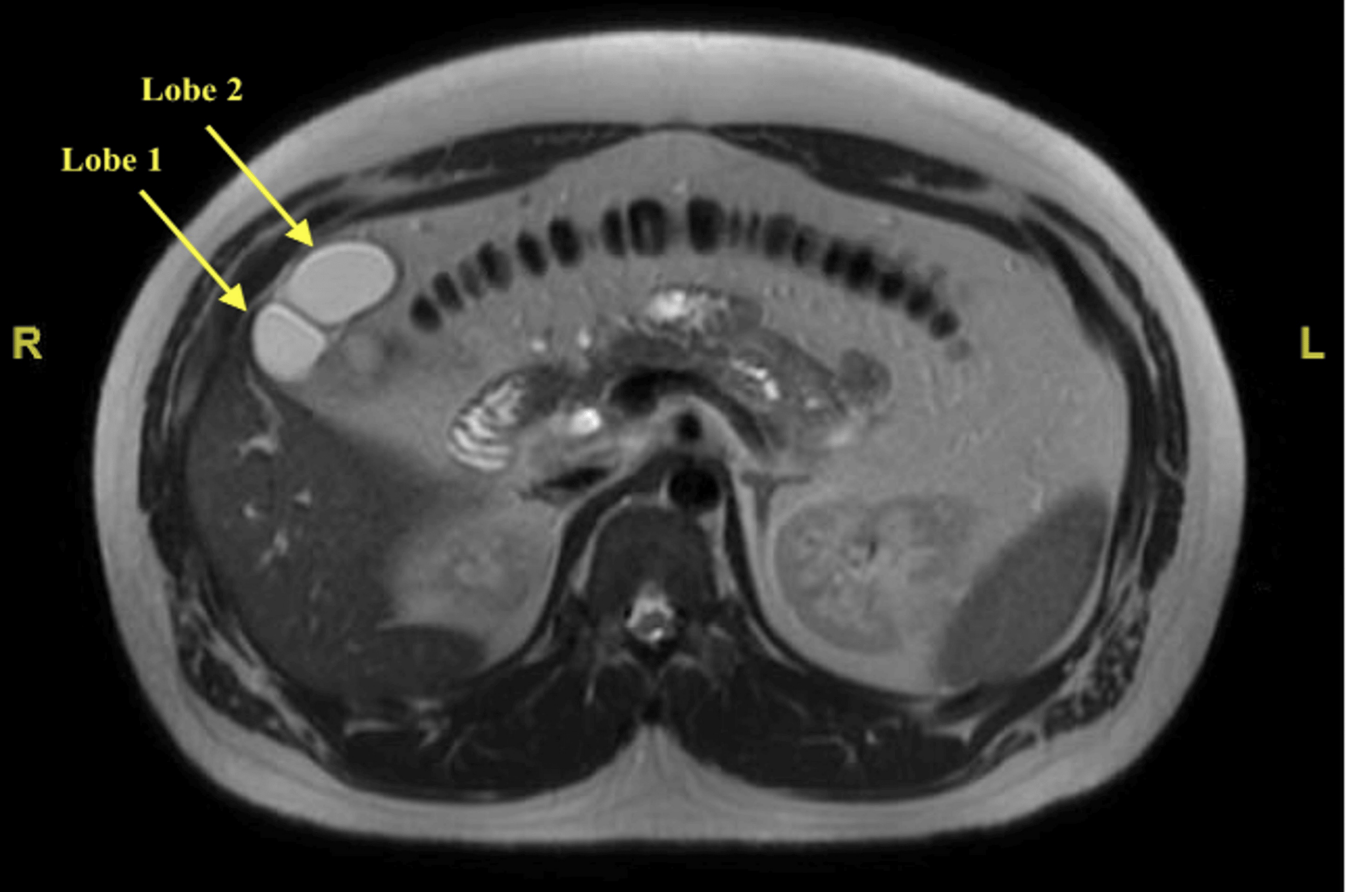
MRCP axial view displaying both lobes of the bilobed gallbladder. MRCP: Magnetic resonance cholangiopancreatography

Access to the abdomen was obtained via 5mm supraumbilical incision and Veress needle insufflation with subsequent 5mm supraumbilical port placement under the Optiview technique. Additional routine laparoscopic ports were placed: two 5 mm right upper quadrant ports and one 12mm epigastric port. Upon visualization of the intra-abdominal structures, two distended sac-like structures were noted within the gallbladder fossa side-by-side with signs of acute or chronic inflammation. The two structures did not appear to communicate with each other on initial evaluation. There was high concern for a choledochal cyst adjacent to the gallbladder, or a duplicated gallbladder, despite no preoperative radiology read for any gallbladder or bile duct abnormalities. MRCP images were evaluated intraoperatively but appeared inconclusive regarding diagnosis. Because of these discordant and unclear findings, an intraoperative radiology consult was obtained to re-evaluate the MRCP images and observe real-time intraoperative laparoscope images for concordance. After extensive discussion with our Radiology colleagues who re-evaluated the MRCP images and evaluated laparoscope images, a determination was made that both distended sac-like structures correlated with the gallbladder, communicated with each other, and attached to the CBD via a single duct, with an intraoperative radiology read of a bilobed gallbladder (Figure 1). No choledochal cyst was noted by our Radiology colleagues.

**Figure 2:**
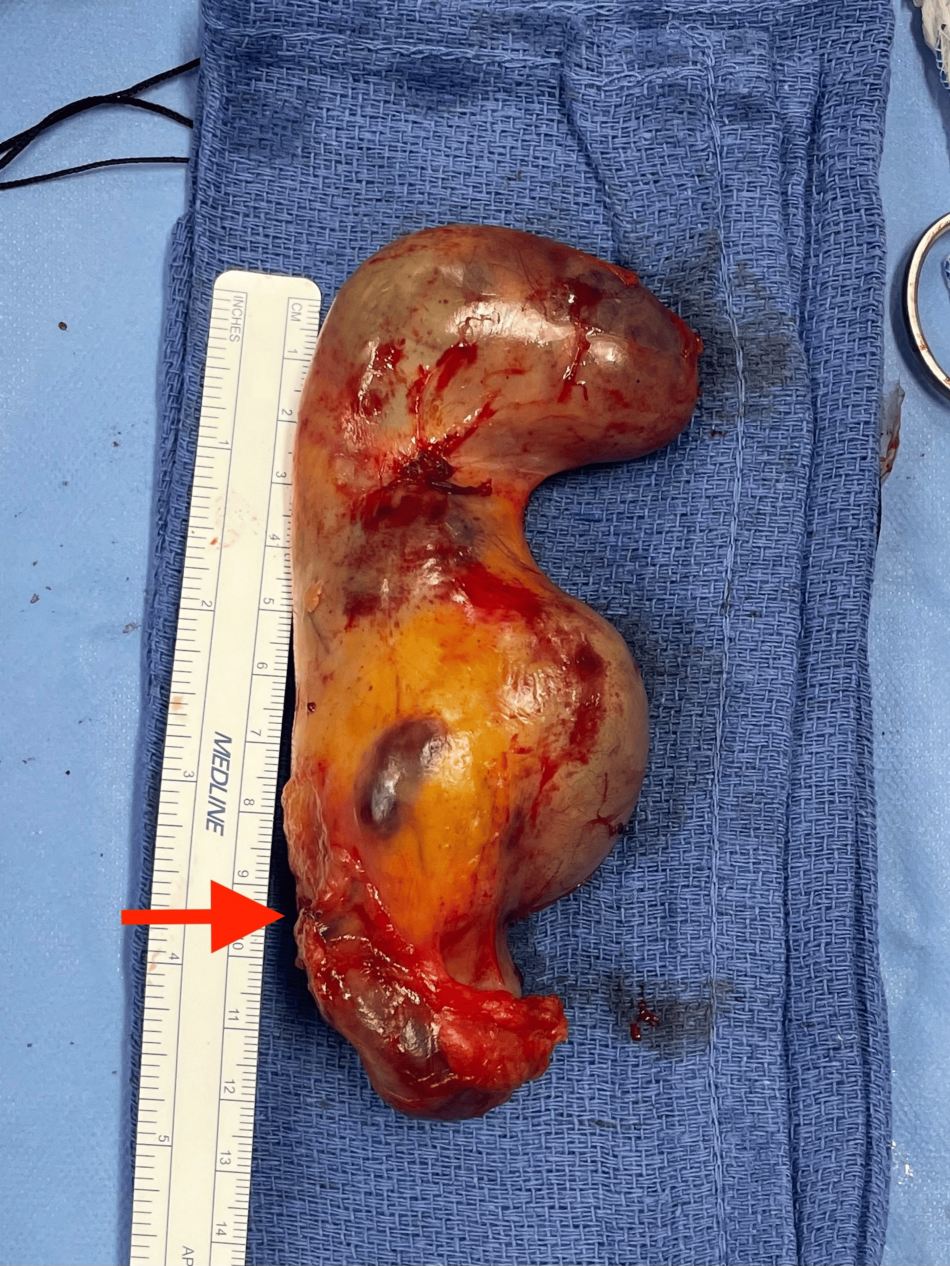
Grossly intact bilobed gallbladder measuring 14 x 5.5 x 4 cm, demonstrating a V-shape with a single cystic duct, with the cystic duct indicated by the red arrowhead.

The decision was made to proceed with laparoscopic cholecystectomy. Classic laparoscopic retraction of the gallbladder fundus toward the right shoulder enables visualization and counter-tension of the hepatocystic triangle and creation of the critical view of safety. However, this was unable to be performed, as both lobes of the gallbladder were adherent to the gallbladder fossa, preventing appropriate retraction. A dome-down approach was used to carefully dissect the entire lateral gallbladder lobe and the majority of the medial gallbladder lobe free. Retraction was then possible, and the critical view of safety was obtained in classic laparoscopic cholecystectomy fashion. The single cystic duct and single cystic artery were clipped and divided, and the remainder of the attached gallbladder was safely dissected off the gallbladder fossa. The gallbladder wall was not compromised at any point, and the bilobed gallbladder was removed intact via a retrieval bag from the epigastric port (Figure 2). There were no intraoperative complications. The postoperative course was uncomplicated. Due to the procedure occurring late in the day, the patient was admitted overnight and discharged the following day. Subsequent surgical pathology confirmed chronic cholecystitis, cholelithiasis, and cholesterolosis with no malignancy, and the patient’s postoperative visit at two weeks revealed well-healing incisions with no complaints.

## Discussion

The gallbladder embryonically arises from the midgut. During this process, duplication of the gallbladder can occur via division or separate origin [2,3,6]. The Harlaftis classification is a widely accepted method of classifying gallbladder duplication [7]. Type 1 embryonically arises via division, comprising two gallbladder lobes with a single cystic duct (Vesica fellea divisa). Type 2 embryonically arises via separate origin, comprising two gallbladder lobes with two cystic ducts (Vesica fellea duplex). Type 1 is rarer than type 2, and bilobed gallbladders are the rarest subset of type 1, classified as Harlaftis type 1b (V-shaped). Type 1a is a septated gallbladder, while type 1c is a Y-shaped double gallbladder [2,3,6,7]. Duplicated gallbladders are not known to cause specific symptoms or a predisposition for gallstone formation [3]. It is unknown whether this association for duplicated gallbladders extends to the bilobed gallbladder subset due to the latter’s rarity. To date, fewer than 30 cases have been reported in the medical literature, and there is scarce information on the medical management and surgical techniques used in these patients.

In the literature, bilobed gallbladders were typically discovered preoperatively on ultrasound or cross-sectional imaging. Open surgery was classically performed for those who required surgery [1], but there are recent case reports detailing laparoscopic management of bilobed gallbladders. A single prior case report documented the intraoperative discovery of bilobed gallbladder during routine laparoscopic cholecystectomy [6]. Diagnosis was accomplished intraoperatively using indocyanine green (ICG) fluoroscopy to evaluate the bile ducts and gallbladder and definitively rule out other causes such as liver cysts or choledochal cysts. This was required due to preoperative computed tomography (CT) imaging that did not reveal the bilobed gallbladder despite repeat postoperative review. In our case, laparoscopic ICG fluoroscopy was not readily available at our institution. However, because we had preoperative MRCP, we were able to obtain an intraoperative radiology consult to re-evaluate the MR cross-sectional and real-time laparoscope imaging for definitive diagnosis of bilobed gallbladder.

If both preoperative cross-sectional imaging and intraoperative ICG fluoroscopy were not available, making diagnosis uncertain, there were two possible options. One would be to obtain an intraoperative endoscopic retrograde cholangiopancreatography (ERCP) to evaluate the ductal system and obtain definitive diagnosis. However, this would require available ERCP services as well as consent, which would likely not have been obtained beforehand. Alternatively, one could abort the procedure and proceed with either MRCP or ERCP with consent to characterize the additional cystic structure before proceeding with the appropriate surgical intervention. Previous reports indicated that ultrasound could be sufficient to diagnose bilobed gallbladder, but our report shows a false negative with ultrasound with diagnosis ultimately obtained by MRCP, which is recommended [2-4]. However, even our preoperative MRCP read was initially interpreted as a distended gallbladder with a narrowed region rather than a bilobed structure. Given that the only other intraoperative diagnosis of bilobed gallbladder [6] also had a false negative for cross-sectional CT imaging both preoperatively and on postoperative re-evaluation, it is not possible to compare sensitivity and specificity of various imaging modalities specifically for diagnosis of bilobed gallbladders to determine which modality is more diagnostic, given the rarity of this phenomenon. Darnis et al. documented sensitivities for imaging modalities for generalized multiple gallbladders (ultrasound 65%, CT 66%, MRCP 97%, ERCP 89%) [2]. However, if a bilobed gallbladder is suspected with negative ultrasound, one should consider MRCP/ERCP to evaluate for such occurrences.

During laparoscopic cholecystectomy for bilobed gallbladders, the bilobed nature of the gallbladder may create difficulty in achieving sufficient exposure and counter tension to obtain critical view of safety for the gallbladder. If that is the case, performing a careful dome-down approach until appropriate exposure is obtained can help facilitate laparoscopic cholecystectomy and avoid conversion to open. If the critical view of safety or adequate dissection are still unable to be obtained despite a dome-down approach, conversion to open cholecystectomy should be considered.

Our case showcases the potential limitations of standard imaging techniques in diagnosing bilobed gallbladders as well as the importance of intraoperative planning and decision-making. In line with previous reports regarding imaging diagnosis [6], our findings suggest that both ultrasound and cross-sectional imaging may have deficiencies in diagnosis. In cases of intraoperative diagnostic uncertainty, preoperative cross-sectional imaging, particularly MRCP or ERCP, as well as intraoperative ICG fluoroscopy should be considered for definitive diagnosis of bilobed gallbladder before proceeding with surgical intervention. Laparoscopic management of bilobed gallbladders is possible, but one should be prepared to use different techniques such as dome-down approach to obtain the critical view of safety. As additional case reports of bilobed gallbladders emerge over time, a consensus on optimal imaging modality for definitive diagnosis will hopefully occur.

## Conclusions

The bilobed gallbladder remains a rare and challenging congenital anomaly with limited case reports documenting its occurrence and management. Our case emphasizes the potential limitations of standard imaging techniques, highlighting the importance of intraoperative decision-making and collaboration with radiology. Laparoscopic cholecystectomy can be successfully performed in cases of bilobed gallbladders, though surgeons must be prepared to adapt their approach, such as utilizing the dome-down technique. However, it is important to recognize that treatment strategies may vary among individual cases, and what is effective in one instance may not be universally applicable. As additional cases are reported, a clearer understanding of optimal diagnostic modalities and surgical techniques will continue to evolve.
